# Probiotic *Lactobacillus rhamnosus* GG Induces Alterations in Ileal Microbiota With Associated CD3^-^CD19^-^T-bet^+^IFNγ^+/-^ Cell Subset Homeostasis in Pigs Challenged With *Salmonella enterica* Serovar 4,[5],12:i:-

**DOI:** 10.3389/fmicb.2019.00977

**Published:** 2019-05-07

**Authors:** Wei Zhang, Qiong Wu, Yaohong Zhu, Guiyan Yang, Jiao Yu, Jiufeng Wang, Haifeng Ji

**Affiliations:** ^1^Institute of Animal Husbandry and Veterinary Medicine, Beijing Academy of Agriculture and Forestry Sciences, Beijing, China; ^2^Animal Science and Technology College, Beijing University of Agriculture, Beijing, China; ^3^Department of Veterinary Clinical Sciences, College of Veterinary Medicine, China Agricultural University, Beijing, China

**Keywords:** *Lactobacillus rhamnosus*, *Salmonella enterica* serovar 4, [5], 12:i:-, gut microbiota, T-bet, IFNγ, pig

## Abstract

*Salmonella enterica* serovar 4,[5],12:i:- (*S.* 4,[5],12:i:-) is an emerging foodborne pathogen causing salmonellosis in humans and animals. Probiotic *Lactobacillus rhamnosus* GG (LGG) is an effective strategy for controlling enteric infections through maintaining gut microbiota homeostasis and regulating the intestinal innate immune response. Here, LGG was orally administrated to newly weaned piglets for 1 week before *S.* 4,[5],12:i:- challenge. *S.* 4,[5],12:i:- challenge led to disturbed gut microbiota, characterized by increased levels of *Psychrobacter, Chryseobacterium indoltheticum*, and uncultured *Corynebacteriaceae* populations, as well as an aberrant correlation network in *Prevotellaceae NK3B31 group*-centric species. The beneficial effect of LGG correlated with attenuating the expansion of *Prevotellaceae NK3B31 group*. *Fusobacterium* only found in the pigs treated with LGG was positively correlated with *Lactobacillus animalis* and *Propionibacterium*. Administration of LGG induced the expansion of CD3^-^CD19^-^T-bet^+^IFNγ^+^ and CD3^-^CD19^-^T-bet^+^IFNγ^-^ cell subsets in the peripheral blood at 24 h after a challenge of *S.* 4,[5],12:i:-. *S.* 4,[5],12:i:- infection increased the population of intraepithelial CD3^-^CD19^-^T-bet^+^IFNγ^+^ and CD3^-^CD19^-^T-bet^+^IFNγ^-^ cells in the ileum; however, this increase was attenuated via LGG administration. Correlation analysis revealed that LGG enriched *Flavobacterium frigidarium* and *Facklamia* populations, which were negatively correlated with intraepithelial CD3^-^CD19^-^T-bet^+^IFNγ^+^ and CD3^-^CD19^-^T-bet^+^IFNγ^-^ cells in the ileum. The present data suggest that probiotic LGG alters gut microbiota with associated CD3^-^CD19^-^T-bet^+^IFNγ^+/-^ cell subset homeostasis in pigs challenged with *S. enterica* 4,[5],12:i:-. LGG may be used in potential gut microbiota-targeted therapy regimens to regulate the specific immune cell function and, consequently, control enteric infections.

## Introduction

The non-typhoidal *Salmonella* (NTS) disease imposes a substantial burden of pediatric and adult morbidity and mortality globally and is estimated to cause 93.8 million human infections and 155,000 deaths annually worldwide (Majowicz et al., 2010; Gilchrist et al., 2015). *Salmonella enterica* (*S. enterica*) serovar 4,[5],12:i:- (*S.* 4,[5],12:i:-) is an emerging foodborne pathogen, causing salmonellosis in domestic and wild animals as well as acute gastroenteritis in humans (Gallati et al., 2013; Mascaro et al., 2017). *S.* 4,[5],12:i:- is believed to be a monophasic variant of *S. enterica* subsp. *enterica* serovar Typhimurium (*S.* Typhimurium) and has lost the genetic basis for encoding the phase 2 flagellin (Ido et al., 2014). The sources of *S.* 4,[5],12:i:- isolates causing foodborne salmonellosis outbreaks in humans often turned out to be pigs and pork products (Rodriguez et al., 2012). In the United States, the prevalence of *S.* 4,[5],12:i:- has increased in the swine industry over the last 20-year period (Yuan et al., 2018). *S.* 4,[5],12:i:- risk to consumers via pork, therefore, poses a considerable threat to public health.

Probiotic *Lactobacillus rhamnosus* GG (LGG) may be an adjunct therapy to attenuate and prevent the course of infection, which will allow more judicious use of prophylactic antibiotics and hopefully reduce overall usage. Several mechanisms underlying the beneficial effects of LGG have been investigated, including competitive exclusion or growth inhibition of pathogens (Petrova et al., 2016; Tytgat et al., 2016), as well as regulation of gut microbiota homeostasis (Berni Canani et al., 2016), or maintaining function of the intestinal barrier (Zhang et al., 2015), and modulation of local or systemic immune response (Johansson et al., 2016). The application of a probiotic combination of *Lactobacillus* strains reduces pathogen shedding and ameliorates disease signs in pigs challenged with *S.* Typhimurium (Casey et al., 2007). Our recent study also found that oral administration of LGG accelerates pathogen clearance and alleviates infection-associated intestinal lesions in weaned piglets challenged with *S.* 4,[5],12:i:- (Yu et al., 2017). The anti-inflammatory capacity of LGG against *S.* 4,[5],12:i:- infection is correlated with inhibition of the canonical nuclear factor-κB pathway and impeding NOD-like receptor family pyrin domain containing 6-mediated inflammasome activation in the intestine (Yu et al., 2017). However, the pathogenesis of *S.* 4,[5],12:i:- and the mechanism underlying the anti-inflammatory activity of LGG against *S.* 4,[5],12:i:- infection remains elusive.

The gut microbiota endows the host with additional functional features, such as pathogen displacement, colonization resistance against pathogens and immune homeostasis. Compared with normal mice, mice harboring a low complexity gut microbiota fail to clear *S*. Typhimurium (Endt et al., 2010). Maintenance of normal gut microbiota dramatically reduces colonization of *S*. Typhimurium (Malo et al., 2010). Using high-throughput sequencing of the 16S ribosomal RNA (rRNA) gene, we recently found that administration with LGG prevents loss of microbial diversity caused by *S. enterica* serovar Infantis and restores the ileal mucosal microbiota balance in newly weaned piglets, thereby restricting systemic *Salmonella* infection (Zhang et al., 2017a). The potential contribution of gut microbiota-targeted LGG to control *S.* 4,[5],12:i:- infection remains unknown.

In the context of intracellular infections, the interactions between gut microbiota and the diverse intestinal immune cells contribute to activation of the immune system as well as to defend against infection. Beside the well-characterized adaptive CD3^+^ T and CD19^+^ B lymphocytes, certain innate non-B-non-T (CD3^-^CD19^-^) cell subsets such as classic cytotoxic nature killer (NK) cells, lymphoid tissue inducer (LTi), and the recently reported non-cytotoxic helper innate lymphoid cells (ILCs) lacking cell-surface molecules, defined as cell lineage marker-negative cells, function synchronously, with the adaptive immune system responding to microbial products or antigens by regulating cytokine secretion (Spits et al., 2013). The T-box transcription factor T-bet is a central regulator of type 1 immunity regulating gamma interferon (IFNγ), which is required for the CD3^-^CD19^-^ cell populations to switch from homeostatic function to potent anti-microbial immunity before the development of adaptive immune responses. Graded expression of T-bet in a type of CD3^-^CD19^-^ cell subset CCR6^-^ RORγt^+^ ILCs induces IFNγ production, aiding the innate defense against *S.* Typhimurium infection in the intestine (Klose et al., 2013). However, *Salmonella* infections cause severe enterocolitis that is at least partly driven by IFNγ (Barthel et al., 2003). We previously reported that LGG consumption regulates T-bet expression in the ileum of pigs, thus ameliorating intestinal inflammation caused by *S. enterica* serovar Infantis (Yang et al., 2017). Limited information is available regarding the mechanism underlying LGG-mediated regulation of the IFNγ-producing CD3^-^CD19^-^ cell subset via T-bet to control *Salmonella* infections.

In the present study, we examined the effect of LGG on the gut microbiota in newly weaned piglets challenged with *S.* 4,[5],12:i:-. We also investigated how LGG regulated the non-B-non-T CD3^-^CD19^-^T-bet^+/-^IFNγ^+/-^ cell subsets during *S.* 4,[5],12:i:- infection.

## Materials and Methods

### Ethics Statement

All animals were treated in strict accordance with the Guidelines for Laboratory Animal Use and Care from the Chinese Center for Disease Control and Prevention and the Rules for Medical Laboratory Animals (1998) from the Chinese Ministry of Health, under protocol CAU20151001-1, which was approved by the Animal Ethics Committee of the China Agricultural University. All surgeries were performed under xylazine hydrochloride anesthesia, and every effort was made to minimize suffering.

### Bacterial Strains

LGG ATCC 53103 (Gefilus, Valio Ltd., Helsinki, Finland) was grown in De Man, Rogosa, and Sharpe (MRS) broth (Oxoid, Basingstoke, United Kingdom) for 24 h at 37°C under microaerophilic conditions. After overnight incubation, the bacteria were inoculated 1:100 in fresh MRS broth and grown for about 8 h until reaching mid-log phase. Bacteria were pelleted by centrifugation at 3,000 × *g* for 10 min at 4°C, washed three times with sterile physiological saline and resuspended in physiological saline. The concentration of LGG was adjusted to 1 × 10^9^ CFU/ml.

The monophasic variant *S. enterica* serovar 4,[5],12:i:- strain (CAU14003) lacking the H antigen *fljB* gene was isolated from the intestinal contents of weaned pigs with diarrhea in our laboratory, as previously described (Yu et al., 2017). The strain was grown to mid-log phase in fresh Luria-Bertani broth (Oxoid) and then was harvested by centrifugation at 6,000 × *g* for 15 min at 4°C, washed three times with sterile physiological saline and resuspended in physiological saline. An inoculum of monophasic variant *S. enterica* serovar 4,[5],12:i:- strain containing 1 × 10^10^ CFU/ml was prepared.

### Experimental Design

The treatment regimens and challenge procedure for the pigs were performed as described elsewhere (Yu et al., 2017). In brief, on the day of weaning (day 0), pigs were assigned to three groups (*n* = 6 per group). Each group received a different treatment, as follows: (1) CN group, oral administration of sterile physiological saline for 7 days; (2) SM group, oral administration of sterile physiological saline for 7 days and oral challenge with monophasic variant *S.* 4,[5],12:i:- on day 8 (1 × 10^10^ CFU/ml, 10 ml); (3) LS group, oral administration of LGG (1 × 10^9^ CFU/ml, 10 ml/day) for 7 days and oral challenge with monophasic variant *S. enterica* serovar 4,[5],12:i:- (1 × 10^10^ CFU/ml, 10 ml) on day 8. At 09:00 am, on days 1 to 7, the pigs in the LS group were intragastrically administrated 10 ml of LGG solution daily, while pigs in the CN and SM groups were pretreated with an equal volume of sterile physiological saline. At 0900 am on day 8, the pigs in groups SM and LS were orally challenged with 10 ml of monophasic variant *S. enterica* serovar 4,[5],12:i:-, whereas the pigs in the CN group received 10 ml of sterile physiological saline. On day 15, the pigs in the three groups were sacrificed.

### Blood and Intestinal Tissue Sampling

Peripheral blood samples were collected from the jugular vein of each pig immediately prior to *Salmonella* challenge (0 h) and at 24 and 168 h after challenge. A 5-ml aliquot of each blood sample containing sodium heparin was analyzed by flow cytometry. The jejunum and ileum segments were harvested immediately after euthanasia. For 16S rRNA gene sequencing, the ileum sections were washed by gently flowing sterile phosphate-buffered saline (PBS) until no contents were visible. The ileal mucosal samples were scraped using a sterile glass microscope slide. All mucosal scraping samples were placed in cryovials, immediately snap-frozen in liquid nitrogen, and then stored at -80°C until DNA extraction. For intestinal mucosal lymphocytes isolation and subsequent flow cytometry analysis, sparse jejunal Peyer’s patches (jPPs), continuous ileal Peyer’s patches (iPPs), and distal jejunum and ileum segments (free of associated PPs) were rinsed with PBS immediately after being opened, divided segmentally, and incubated in Hanks’ balanced salt solution (HBSS; 10 mM HEPES, 50 U/ml penicillin, 50 μg/ml streptomycin) lacking Ca^2+^ and Mg^2+^.

### *Salmonella* Enumeration

One gram of fresh fecal or ileal mucosal sample from each animal was homogenized in 9 ml of sterile saline solution, and serial dilutions were then plated on xylose lysine sodium deoxidized acid agar medium for 24 h at 37°C under aerobic conditions for selection and enrichment of *Salmonella*.

### Genomic DNA Extraction and PCR Amplification

Total bacterial genomic DNA was extracted using a QIAamp DNA Microbiome kit (Qiagen, Hilden, Germany) according to the manufacturer’s instructions. This kit allows the efficient depletion of animal host DNA. The V3–V4 region of the bacterial 16S rRNA gene were amplified by PCR (95°C for 3 min, followed by 27 cycles at 95°C for 30 s, 55°C for 30 s, and 72°C for 45 s and a final extension at 72°C for 10 min) using primers 338F (5′-ACT CCT ACG GGA GGC AGC AG-3′) and 806R (5′-GGA CTA CHV GGG TWT CTA AT-3′), where barcode was an eight-base sequence unique to each sample. PCR reactions were performed in triplicate 20 μl of mixture (TransGen Biotech, Beijing, China), containing 4 μl of 5 × FastPfu Buffer, 2 μl of 2.5 mM dNTPs, 0.8 μl of each primer (5 μM), 0.4 μl of FastPfu Polymerase, and 10 ng of template DNA.

### Quantification of *L. rhamnosus* by qPCR

Quantification of *L. rhamnosus* 16S rRNA genes was performed using an ABI 7500 real-time PCR system (Applied Biosystems, Foster City, CA, United States), as previously described (Zhang et al., 2017b). The following specific primer sets for *L. rhamnosus* species were used: 5′-CGG CTG GAT CAC CTC CTT T-3′, 5′-GCT TGA GGG TAA TCC CCT CAA-3′ (Haarman and Knol, 2006). Each run included a standard curve and each sample was run in triplicate. Bacterial contents were interpolated from a standard curve and then weight corrected to yield a value in 16S rRNA gene copy number/g sample. Samples, standards, and non-template controls were run in triplicate.

### 16S rRNA Gene Sequencing

Amplicons were extracted from 2% agarose gels and purified using the AxyPrep DNA Gel Extraction Kit (Axygen Biosciences, Union City, CA, United States) according to the manufacturer’s instructions and quantified using QuantiFluor^TM^ ST (Promega, Madison City, WI, United States). Purified amplicons were pooled in equimolar and paired-end sequenced (2 × 300 bp) on an Illumina Miseq platform according to the standard protocols.

### Phylotype Analysis

The raw sequence data were processed and demultiplexed using the Trimmomatic and Quantitative Insights Into Microbial Ecology (QIIME) pipeline^1^. Pairs of reads from the original DNA fragments were merged using FLASH and assigned to their respective sample according to barcodes. Raw fastq files were quality-filtered using QIIME with the criteria as previously described (Zhang et al., 2017a,b). Chimeras were checked and excluded against the Silva Gold reference database ^2^ using Uchime algorithm. The resulting high-quality reads were clustered into the operational taxonomic unit (OTU) using a closed-reference OTU picking protocol with Usearch 6.1 methodology (version v6.1.544) being applied to search sequences against a subset of the Silva 16S sequences database filtered at a pairwise distance of 97% sequence identity. Reads that did not match a reference sequence were discarded. The most abundant sequences from each OTU were selected as representative sequence and were assigned to taxonomic classification against the Silva (SSU117/119) database clustered at a bootstrap cutoff of 80% via the Ribosomal Database Project naive Bayesian classifier^3^. Prior to computing diversity distances, samples were randomly subsampled down to the lowest number of reads. Alpha diversity metrics (Shannon), richness (ACE and Chao1), Good’s coverage and rarefaction analysis were calculated to assess bacterial diversity using Mothur version 1.31.2^4^. Data analysis were performed using the online platform of Majorbio I-Sanger Cloud Platform ^5^.

Heatmap and network analysis were performed to visualize the taxon abundance at the genus level using Cytoscape version 2.8^6^ and R version 3.2.1^7^, respectively. The linear discriminant analysis (LDA) effect size (LEfSe) method was used to identify indicator bacteria that distinguished the treatment-specific microbiota features. Multivariate data analysis, including Venn analysis of shared and unique OTUs and partial least squares-discriminate analysis (PLS-DA), was performed using R and Simca-*P* 12.0 (Umetrics, Umea, Sweden), respectively.

### Co-occurrence Network Analysis

Pearson’s correlations or associations between all OTUs were calculated. Each OTU’s abundance was normalized by log-transformation. Statistic *P*-values were corrected using the Benjamini-Hochberg’s false discovery rate (FDR, *q*-value) method. Correlations with an absolute Pearson’s correlation above 0.7 and FDR-corrected significance level under 0.05 were transformed into links between two OTUs in the OTU co-occurrence network. The co-occurrence networks were then visualized using Cytoscape version 2.8.2 with a force-directed algorithm (Smoot et al., 2011), and network topological parameters were determined using Networks (Assenov et al., 2008). The nodes in this network represented OTUs and the edges that connected these nodes represented correlations between OTUs.

### Isolation of Peripheral Blood Lymphocytes

Peripheral blood lymphocytes were obtained by Ficoll gradient centrifugation using lymphocyte separation solution (TBD Science Inc., Tianjin, China), according to the manufacturer’s instructions. In brief, 2 ml of peripheral blood samples were diluted with an equal volume of diluent and then were gently added in 4 ml of Ficoll solution. Lymphocytes were stratified over Ficoll solution by centrifugation at 400 × *g* for 20 min at room temperature. Recovered lymphocytes were washed three times with RPMI 1640 medium (Gibco, Grand Island, NY, United States). The living lymphocytes were counted using trypan blue staining method and were used for flow cytometry analysis.

### Isolation of Intestinal Mucosal Lymphocytes

Intestinal mucosal lymphocytes, including Peyer’s patch lymphocytes (PPLs), intraepithelial lymphocytes (IELs), and lamina propria lymphocytes (LPLs) were isolated as previously described (Zhu et al., 2014). In brief, to isolate IELs, Peyer’s patched-free tissues of 3-cm in length were transferred into Ca^2+^- and Mg^2+^- free HBSS containing 2 mM dithiothreitol (DTT) and 10 mM HEPES (HBSS-DTT) for 5 min at 37°C. Then, the samples were incubated with Ca^2+^- and Mg^2+^- free HBSS containing 3 mM EDTA and 10 mM HEPES (HBSS-EDTA) by gently shaking for 45 min at 37°C. After passing through a sterile 200-μm-pore metal sieve, the cells pellet was harvested by centrifugation at 600 × *g* for 10 min at 4°C, resuspended in RPMI 1640 medium and kept on ice. The remaining tissue was thoroughly washed with RPMI 1640 medium, followed by three times digestion with RPMI 1640 containing 100 U/ml collagenase VII (Sigma-Aldrich, Saint Louis, MO, United States) for 45 min at 37°C in an orbital shaker. The resulting LPLs were filtered through a sterile 200-μm-pore metal sieve and then centrifuged at 600 × *g* for 10 min at 4°C. The IELs and LPLs were purified using 25% to 44% to 66% Percoll gradient centrifugation (GE Healthcare, Piscataway, NJ, United States). The PPLs were isolated from tissues after incubation with HSBB-DTT and digestion with HBSS-EDTA. Then, the cells were harvested through gentle mincing in a petri dish containing HBSS, followed by filtering and centrifugation as described above. The resulting PPLs were purified and collected from the interface between 40 and 70% Percoll layers.

### Flow Cytometry

For flow cytometry analysis, the following antibodies were used: fluorescein isothiocyanate [FITC]-conjugated mouse anti-pig CD3ε (clone P2G10, 559582), PerCP-Cy5.5-conjugated mouse anti-human CD19 (clone HIB19, 561295), phycoerythrin [PE]-conjugated mouse anti-human T-bet (clone O4-46, 561268), and AlexaFluor 647-conjugated mouse anti-pig IFNγ (clone P2G10, 561480), along with isotype control FITC-conjugated mouse IgG2a, κ (553390), PE-conjugated mouse IgG1, κ (559320), AlexaFluor 647-conjugated mouse IgG1, κ (557732) and PerCP-Cy5.5-conjugated mouse IgG2b, κ (558304). All antibodies were purchased from BD Biosciences (San Jose, CA, United States).

Lymphocytes isolated from peripheral blood and intestines were stimulated in complete RPMI 1640 medium containing 50 ng/ml of phorbol 12-myristate 13-acetate (PMA; Sigma), 5 mM calcium ionomycin A23187 (Sigma), and Golgistop (BD Biosciences) for 4 h at 37°C. Stimulation of PMA/ionomycin at this concentration did not affect the expression of surface marker CD3. After this *in vitro* stimulation, cells were washed with staining buffer (BD Biosciences) and surface CD3 and CD19 were stained with FITC-conjugated mouse anti-pig CD3ε and PerCP-Cy5.5-conjugated mouse anti-human CD19 for 30 min at 4°C. For intracellular staining, cells were fixed and permeabilized with BD Cytofix/Cytoperm Fixation/Permeabilization Kit (BD Biosciences) and stained with PE-conjugated mouse anti-human T-bet and AlexaFluor 647-conjugated mouse anti-pig IFNγ for 30 min at 4°C. Isotype controls with irrelevant specificities were used as negative controls. Data were collected on a FACScalibur flow cytometer (BD Biosciences) and were analyzed using FlowJo 9.3 software (Tree Star, Ashland, OR, United States).

### Accession Number

The raw 16S rRNA reads were deposited in the US National Center for Biotechnology Information Sequence Read Archive database (Accession Number: SRP364367).

### Statistical Analysis

Statistical predictors of community composition were tested based on the Bray–Curtis distance using a permutational multivariate analysis of variance (ADONIS) test implemented in QIIME. *P*-value was calculated using 999 permutations. LEfSe was performed to discover indicator bacteria that distinguished the treatment-specific microbiota features, and LDA was used to estimate the effect size of each feature. A significance level (alpha) of 0.05 and an effect size threshold of 2 was used for all indicators discussed in this study.

Statistical analysis of the relative abundances of taxa was performed using the SAS statistical software package, version 9.3 (SAS Institute Inc., Cary, NC, United States). The UNIVARIATE (Shapiro–Wilk test) and HOVTEST procedures were used to test normal distribution and homogeneity of variance. Normalized data were tested using PROC MIXED procedures followed by Tukey’s honestly significant difference *post hoc* test. Non-normally distributed data were analyzed using the PROC GLIMMIX procedures. A *P*-value of <0.05 with a *q*-value of <0.05 was considered indicative of significance. The CD3^-^CD19^-^T-bet^+/-^IFNγ^+/-^ cell data were analyzed using the PROC MIXED procedure. For data from blood samples, the statistical model included the fixed effects of treatment, litter, sex, sampling time, interactions between treatments and sampling time, as well as the random effect of individual pigs within a treatment. In addition, a first-order autoregressive covariance structure was applied to the model to account for the correlation between measures at different times within individual pigs. For data from intestinal tissue, the statistical model included the fixed effects of treatment, litter, sex, intestinal section, interactions between treatments and intestinal section, as well as random effects associated with individual pigs within a treatment. Differences between least-square means were compared using Tukey’s honestly significant difference *post hoc* test. Differences were considered significant when *P* < 0.05. Correlations between OTU abundances and CD3^-^CD19^-^T-bet^+/-^IFNγ^+/-^ cell subsets were identified using Spearman’s correlation implemented in R. Correlations having a *P*-value of <0.05 with a *q*-value of <0.05 was considered significant.

## Results

### Clinical Status

Before the challenge, all pigs exhibited a normal rectal temperature (39.3 ± 0.32°C). Compared with the CN group, from 6 to 72 h after *S.* 4,[5],12:i:- challenge, pigs in the SM group showed a continued high rectal temperature (41.2 ± 0.15°C, *P* < 0.001). Although pigs in the LS group had a higher rectal temperature (more than 40.0°C, *P* = 0.011) than pigs in the CN group at 6 h after *S.* 4,[5],12:i:- infection, the rectal temperature of pigs in the LS group returned to normal at 48 h after challenge.

The incidence of diarrhea was recorded daily throughout the experiment. During week 1 (prior to challenge), only 1 pig in the CN group exhibited naturally acquired mild diarrhea lasting for 2 days due to weaning stress. During week 2, two pigs in the SM group and three pigs in the LS group exhibited temporarily mild and serious watery diarrhea lasting for 1 day following challenge. No differences were observed in the diarrhea score distributions in week 1 and week 2 among the three groups. When presented as pig-days with diarrhea, the incidence of diarrhea was unaffected by the challenge.

No differences were observed with respect to average daily gain at 2 weeks (161, 123, and 149 g/d for CN, SM, and LS groups, respectively; *P* > 0.05) between each group. Similarly, neither the average daily feed intake nor gain-to-feed ratio was not changed by treatments.

### Measurement of *Salmonella* and *L. rhamnosus*

Fecal shedding of *Salmonella* was measured using the plate counting method. Pigs in the LS (3.86 × 10^3^ ± 2.52 × 10^3^ CFU g^-1^ of feces [mean ± SD]) and SM (3.53 × 10^3^ ± 1.39 × 10^3^ CFU g^-1^ of feces) groups began to shed *Salmonella* at 6 and 12 h after challenge, respectively, and excreted *Salmonella* in their feces until the end of the experiment. Compared to the SM group, the amount of *Salmonella* shed was higher in the LS group at 12 h after challenge. The amount of *Salmonella* in the ileal mucosal samples was unchanged among the SM and LS groups. Fecal and ileal samples collected from pigs in the CN group were negative for *Salmonella* throughout the experiment, indicating the detected *Salmonella* in the SM and LS groups was *S.* 4,[5],12:i:-.

The abundance of *L. rhamnosus* was measured using quantitative PCR. Compared to the CN (3.15 × 10^2^ ± 1.12 × 10^2^ copy number g^-1^ of ileal mucus [mean ± SD]) and SM (3.56 × 10^2^ ± 2.28 × 10^2^ copy number g^-1^ of ileal mucus) pigs, the LS pigs had higher abundance of *L. rhamnosus* (4.72 × 10^3^ ± 2.45 × 10^3^ copy number g^-1^ of ileal mucus). No changes in the abundance of *L. rhamnosus* were observed between the CN and SM groups.

### High-Throughput Sequencing and Quality Control

A raw dataset consisting of 329,418 reads and 132,526,281 bp was yielded. After quality trimming and chimera checking, a total of 294,876 high-quality 16S rRNA gene reads were obtained from the 18 samples, with an average of 16,382 ± 2,143 reads (means ± standard deviation) per sample. No significance of sequence numbers among all samples indicated a minimal sequencing bias. The length of 294,870 high-quality reads was between 401 and 500 bp, with a median sequence read length of 449 bps. At this sequencing depth, the individual rarefaction curves tended to approach the saturation plateau (Supplementary Figure S1A), but Shannon diversity curves for all samples were stable (Supplementary Figure S1B). This suggested that although rare new phylotypes would still appear upon further sequencing, most diversity had already been covered. The rank abundance curves exhibited a sharp decline and a long tail (Supplementary Figure S1C), indicating that a small number of OTUs were dominated and the majority of OTUs were present at low abundance in the ileal mucosal microbiota. Across all samples, a total of 123 bacteria OTUs were identified, with an average of 87 ± 6 OTUs per sample. No differences in the number of OTUs were observed among three groups, indicating there was no different in richness between the sampled communities.

### Characterization of the Ileal Mucosal Microbiota of Pigs

**FIGURE 1:**
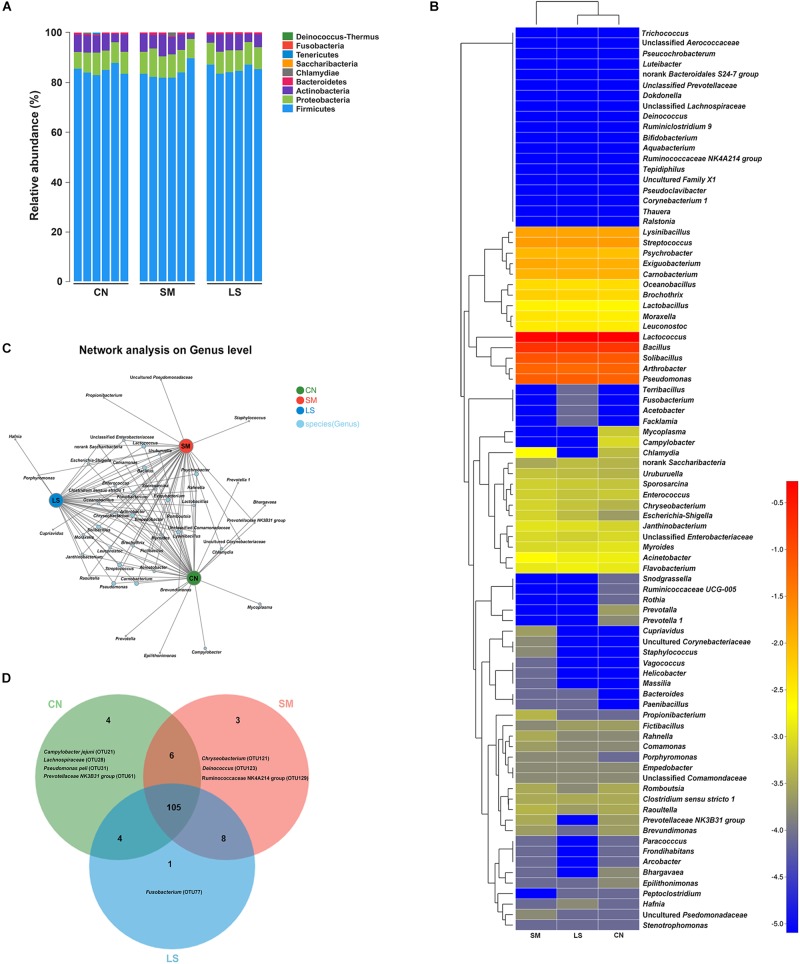
Ileal mucosal microbiota communities of newly weaned pigs in response to *S.* 4,[5],12:i:- challenge and LGG pretreatment. **(A)** Ileal mucosal microbiota communities at the phylum level. The stacked bars show the combined relative abundance of phylum-level taxa per animal. Colors are assigned for all phyla detected. **(B)** Heatmap showing the spatial distributionsof all OTUs at the genus level, indicating the relative abundance of genera per animal (*n* = 6 per group) that received oral sterile physiological saline (CN), oral sterile physiological saline followed by *S.* 4,[5],12:i:- (1 × 10^10^ CFU/ml, 10 ml) challenge (SM), or LGG (1 × 10^9^ CFU/ml, 10 ml/day) for 1 week followed by *S.* 4,[5],12:i:- (LS). Genera are clustered to the left based on relative abundance. The relative abundance of each genus is indicated by a color gradient from blue (low abundance) to red (high abundance). **(C)** A network diagram showing the OTUs among the three groups. **(D)** Venn diagrams displaying the overlap degree of OTUs in the ileal mucosal microbiota of the three groups.

Taxon-based analysis revealed that the ileal mucosal microbiota profiles at the phylum level were congruent among three groups and nine phyla were identified. *Firmicutes* populations, accounting for 85% of all the sequences, were predominant in the ileal mucosal microbiota, regardless of treatments (Figure 1A). In addition, *Proteobacteria, Actinobacteria, Bacteroidetes*, and *Saccharibacteria* were present in all samples and constituted 8.23%, 6.15%, 0.39% and 0.05% of all the sequences, respectively. Four other phyla were identified, with three, four and two phyla in the CN, SM and LS groups, respectively. For example, *Chlamydiae* was absent in the LS group. *Deinococcus-Thermus* was only found in one pig in the SM group and *Tenericutes* was present in one pig in the CN and SM groups, respectively. The relative abundances of taxa in the phylum level did not differ among three groups.

In total, usable sequences were classified into 86 genera. *Lactococcus piscium* (OTU57) belonging to *Firmicutes* accounted for 45% of all the sequences. The 13 other most abundant taxa (with a mean relative abundance > 1%) accounted for 49% of all the sequences (Supplementary Table S1), comprising *Lactococcus* (OTU66, 8.08%), *Solibacillus* (OTU18, 8.17%), *Bacillus* (OTU19 [6.74%], OTU85 [6.36%], OTU67 [2.08%] and OTU50 [1.49%]), *Lysinibacillus* (OTU89, 1.50%), *Exiguobacterium* (OTU46, 1.24%) and *Carnobacterium* (OTU107, 1.11%) belonging to *Firmicutes, Pseudomonas* (OTU86 [2.67%], OTU68 [2.01%] and OTU41 [1.9%]) belonging to *Proteobacteria*, and *Arthrobacter* (OTU108, 5.71%) belonging to *Actinobacteria*. The heatmap-based analysis at genera level showed the microbiota profiles of pigs in the LS group was similar to that in the CN group (Figure 1B).

The predominant genera co-occurred and the abundance of some rare genera differed in three groups. The genera *Prevotella, Prevotella 1, Epilithonimonas, Campylobacter, Bhargavaea* and *Mycoplasma* were unique in the CN group. *Salmonella* infection enriched the genera *Porphyromonas, Propionibacterium, Staphylococcus, Cupriavidus* and an uncultured *Pseudomonadaceae* (Figure 1C). *Hafnia* was enriched in the LS group. The taxon-independent Venn analysis showed that 105 OTUs were shared by the three groups, accounting for 80.15% of all OTUs. Unique OTUs accounted for 0.12%, 0.0098% and 0.012% of all the sequences and 3.05%, 2.29% and 0.0076% of the total OTUs in the CN, SM and LS groups, respectively (Figure 1D). *Chryseobacterium* (OTU121), *Deinococcus* (OTU123) and *Ruminococcaceae NK4A214 group* (OTU 128) were only found in the SM group. *Fusobacterium* (OTU77) was unique in the LS group.

### LGG Pretreatment Affected the Ileal Mucosal Microbiota Structure During *Salmonella* Infection

**FIGURE 2:**
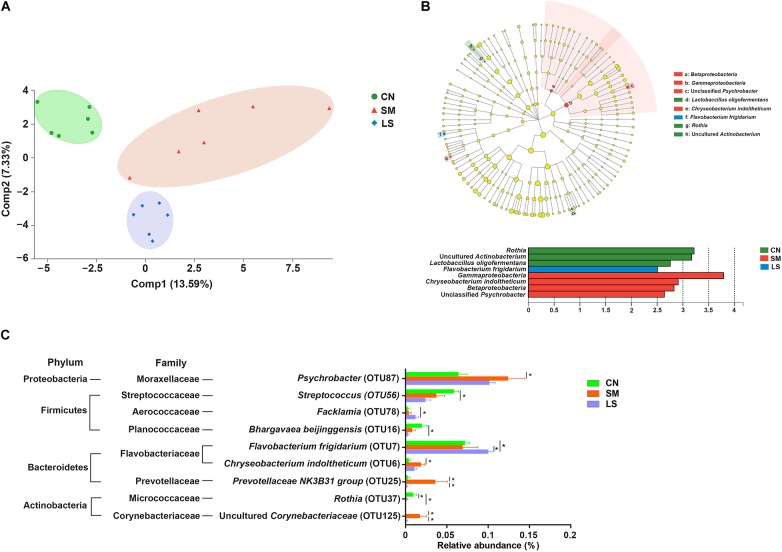
LGG pretreatment changed the ileal mucosal microbiota structure of pigs challenged with *S.* 4,[5],12:i:-. **(A)** Based on the relative abundance of OTUs, two-dimensional PLS-DA score plots distinguishing the ileal mucosal microbiota of pigs in response to *S.* 4,[5],12:i:- challenge and LGG pretreatment. **(B)** LEfSe was performed to discover indicator bacteria that distinguished the treatment-specific microbiota features. Taxonomic cladogram indicating the phylogenetic distribution of microbial lineages associated with the three groups (*n* = 6 per group). Lineages with an LDA value >2.5 are displayed. The diameter of each dot is proportional to its effect size. **(C)** Differences in relative abundance of OTUs from the three indicated treatment groups are shown using one-way ANOVA. The taxonomy of the OTUs (phylum and family) is depicted on the left. ^∗^*P* < 0.05.

The overall structure of the ileal mucosal microbiota, considered at OTU level as well as taxonomic levels ranging from genus to phylum, was significantly altered by the treatments, as determined by ADONIS (*P* < 0.05 for all levels, Supplementary Table S2). The PLS-DA based on the relative abundance of OTUs showed clearly distinguishable ileal mucosal microbiota samples among the three groups (Figure 2A).

LEfSe results showed that in the CN group, two lineages had an LDA value of 2.5 or higher, namely, an uncultured *Actinobacterium* within the genus *Rothia*, and *Lactobacillus oligofermentans* (Figure 2B). Compared with the CN group, *Betaproteobacteria* populations, an unclassified *Psychrobacter* genus within *Gammaproteobacteria* and *Chryseobacterium indoltheticum* were increased in the SM group, with an LDA value > 2.5, while *Flavobacterium frigidarium* was enriched in the LS group. Further, one-way ANOVA identified the LEfSe results. Compared with the CN group, the relative abundances of *Psychrobacter, Chryseobacterium indoltheticum, Prevotellaceae NK3B31 group*, and an uncultured *Corynebacteriaceae* were increased in the SM group, and the relative abundance of *Rothia* was decreased in the SM and LS groups (Figure 2C). However, LGG pretreatment attenuated the *Salmonella*-induced increase in the relative abundance of *Prevotellaceae NK3B31 group* and an uncultured *Corynebacteriaceae*. Compared with the CN and SM groups, the relative abundance of *Flavobacterium frigidarium* was increased in the LS group.

### Co-occurrence Network of Ileal Mucosal Microbiota in Response to *Salmonella* Challenge and LGG Pretreatment

**FIGURE 3:**
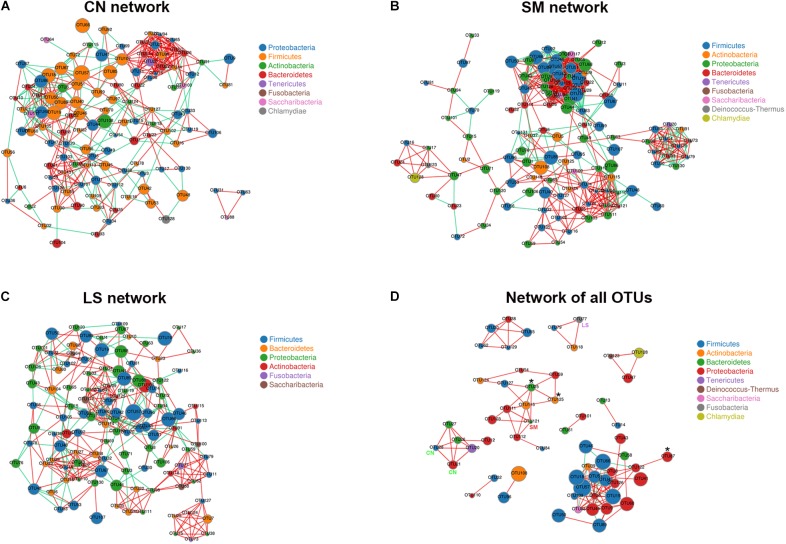
Co-occurrence networks of microbiota associated with *S.* 4,[5],12:i:- challenge and LGG pretreatment. Networks were constructed using OUT pairs present in the microbiota of pigs in the CN **(A)**, SM **(B)**, and LS **(C)** groups, as well as across the three groups **(D)**, all with an absolute Pearson’s correlation above 0.7 at a 0.05 FDR-corrected significance level. OTUs are colored by phylum affiliation and sized by mean relative abundance. In **(D)**, asterisk (^∗^) represents OTUs whose abundances were significantly altered among the three groups. CN, SM, and LS represent OTUs who were only found in the CN, SM, and LS group, respectively.

A correlation network analysis using OTUs was performed to evaluate if *Salmonella* infection and LGG pretreatment were associated with changes in the correlation structure and putative interaction structure of the ileal microbiota. Compared with that in the CN group (Figure 3A), network in the SM group had a higher mean degree, more significant correlations and more clustering of OTUs (Figure 3B and Supplementary Table S3). The network in the LS group was similar to that in the CN group, with a fewer mean degree and less clustering of OTUs (Figure 3C and Supplementary Table S3).

A co-occurrence network of all observed OTUs across all samples captured 114 positive correlations (that is, symbiotic relationships between species) and 18 negative correlations (that is, colonization resistance or oppositional relationships between species) (Figure 3D and Supplementary Table S4). The network contained 60 nodes and 132 edges. Ten consortia of interactive OTUs that were composed of two to 23 nodes were observed. OTU19 (*Bacillus*) had the most linkers (19 degrees). The network contained some OTUs specific to treatment and OTUs with differential abundances among the three groups. Both OTU21 (*Campylobacter jejuni*) and OTU28 (an unclassified genus within the family *Lachnospiraceae*) specific to the CN group were positively correlated with OTU12 (*Brevundimonas*), OTU20 (*Mycoplasma hyorhinis*), OTU26 (*Prevotella 1*), and OTU27 (*Prevotella*). Differential OTU25 was positively correlated with OTU111 (*Acinetobacter*), OTU121 (*Chryseobacterium*, specific to the SM group), OTU125 (an uncultured genus within the family *Corynebacteriaceae*, significantly increased in the SM group) and OTU127 (*Staphylococcus*). OTU87 (*Psychrobacter*) enriched in the SM group was positively correlated with OTU122 (*Rahnella*). OTU77 (*Fusobacterium*) specific to the LS group was positively correlated with OTU79 (*Lactobacillus animalis*) and OTU118 (*Propionibacterium*).

### LGG Pretreatment Induced the Expansion CD3^-^CD19^-^T-bet^+^IFNγ^+^ and CD3^-^CD19^-^T-bet^+^IFNγ^-^ Cell Subsets in the Peripheral Blood

**FIGURE 4:**
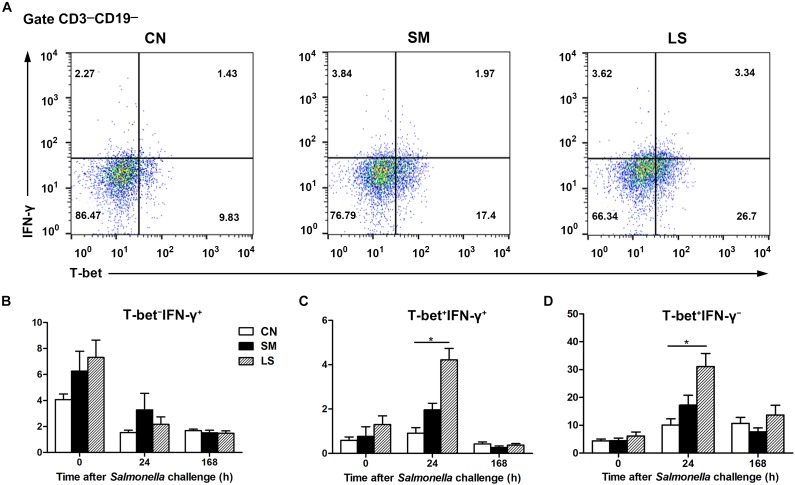
LGG Pretreatment induced the expansion CD3^-^CD19^-^T-bet^+^IFNγ^+^ and CD3^-^CD19^-^T-bet^+^IFNγ^-^ cell subsets in the peripheral blood. Peripheral blood samples were collected from the indicated pigs at 0, 24, and 168 h after *S.* 4,[5],12:i:- challenge. **(A)** Representative dot plots show the percentages of T-bet^+/-^ IFNγ^+/-^ cell subsets among CD3^-^CD19^-^ cells at 24 h after *S.* 4,[5],12:i:- challenge. Flow cytometry analysis of the percentages of T-bet^-^ IFNγ^+^ **(B)**, T-bet^+^ IFNγ^+^ **(C)**, and T-bet^+^ IFNγ^-^ cells **(D)** within the peripheral CD3^-^CD19^-^ cell populations in the indicated pigs. The data are presented as mean ± SEM (*n* = 6 per group). ^∗^*P* < 0.05.

To explore the role of the transcription factor T-bet in regulating IFNγ expression, CD3^-^CD19^-^T-bet^+/-^IFNγ^+/-^ cell subsets were examined. Gating strategy for cell analysis by flow cytometry in the peripheral blood was shown in Supplementary Figure S2. Representative results were shown in Figure 4A. Interestingly, at 24 h after *Salmonella* challenge, compared with the CN pigs, LGG pretreatment induced an increase in the percentage of CD3^-^CD19^-^T-bet^+^IFNγ^-^ and CD3^-^CD19^-^T-bet^+^IFNγ^-^ cells in the peripheral blood (Figure 4C,D). No changes were observed in the percentage of CD3^-^CD19^-^T-bet^-^IFNγ^-^ cells in the peripheral blood, regardless of treatments (Figure 4B).

### LGG Pretreatment Attenuated *Salmonella*-Induced Expansion of Intraepithelial CD3^-^CD19^-^T-bet^+^IFNγ^+^ and CD3^-^CD19^-^T-bet^+^IFNγ^-^ Cell Subsets in the Ileum

We assessed changes in the proportion of T-bet^+/-^IFNγ^+/-^ cell subsets among CD3^-^CD19^-^ cell populations in the intestinal compartments, including the PPs, intraepithelial layer, and the lamina propria of the jejunum and ileum.

**FIGURE 5:**
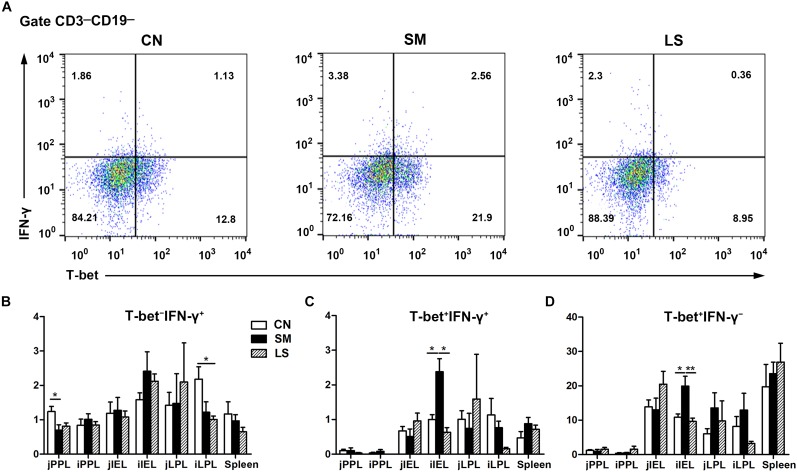
LGG Pretreatment Attenuated *Salmonella*-induced expansion of intraepithelial CD3^-^CD19^-^T-bet^+^IFNγ^+^ and CD3^-^CD19^-^T-bet^+^IFNγ^-^ cell subsets in the ileum. Peyer’s patch lymphocytes (PPLs), intraepithelial lymphocytes (IELs), and lamina propria lymphocytes (LPLs) were collected from jejunal and ileal tissues from the indicated pigs 7 days after *S.* 4,[5],12:i:- challenge. **(A)** Representative dot plots show the percentages of intraepithelial T-bet^+/-^ IFNγ^+/-^ cell subsets among CD3^-^CD19^-^ cells. Flow cytometry analysis of the percentages of T-bet^-^ IFNγ^+^ **(B)**, T-bet^+^ IFNγ^+^ **(C)**, and T-bet^+^ IFNγ^-^ cells **(D)** among CD3^-^CD19^-^ cell populations in the small intestine. The data are presented as mean ± SEM (*n* = 6 per group). ^∗^*P* < 0.05, ^∗∗^*P* < 0.01.

Gating strategy for cell analysis by flow cytometry in the intestines was shown in Supplementary Figure S3. Representative results were shown in Figure 5A. Compared with the CN pigs, a decrease in the percentage of CD3^-^CD19^-^T-bet^-^IFNγ^+^ cells was observed among jPPL from the SM pigs and among iLPL from the LS pigs, respectively (Figure 5B). The proportions of CD3^-^CD19^-^T-bet^+^IFNγ^+^ (Figure 5C) and CD3^-^CD19^-^T-bet^+^IFNγ^-^ (Figure 5D) cell subsets were increased among iIEL from the SM pigs, but the increased was attenuated by LGG pretreatment (*P* < 0.05).

### Correlation Between OTUs and CD3^-^CD19^-^T-bet^+/-^IFNγ^+/-^ Cell Subsets in the Ileum

**FIGURE 6:**
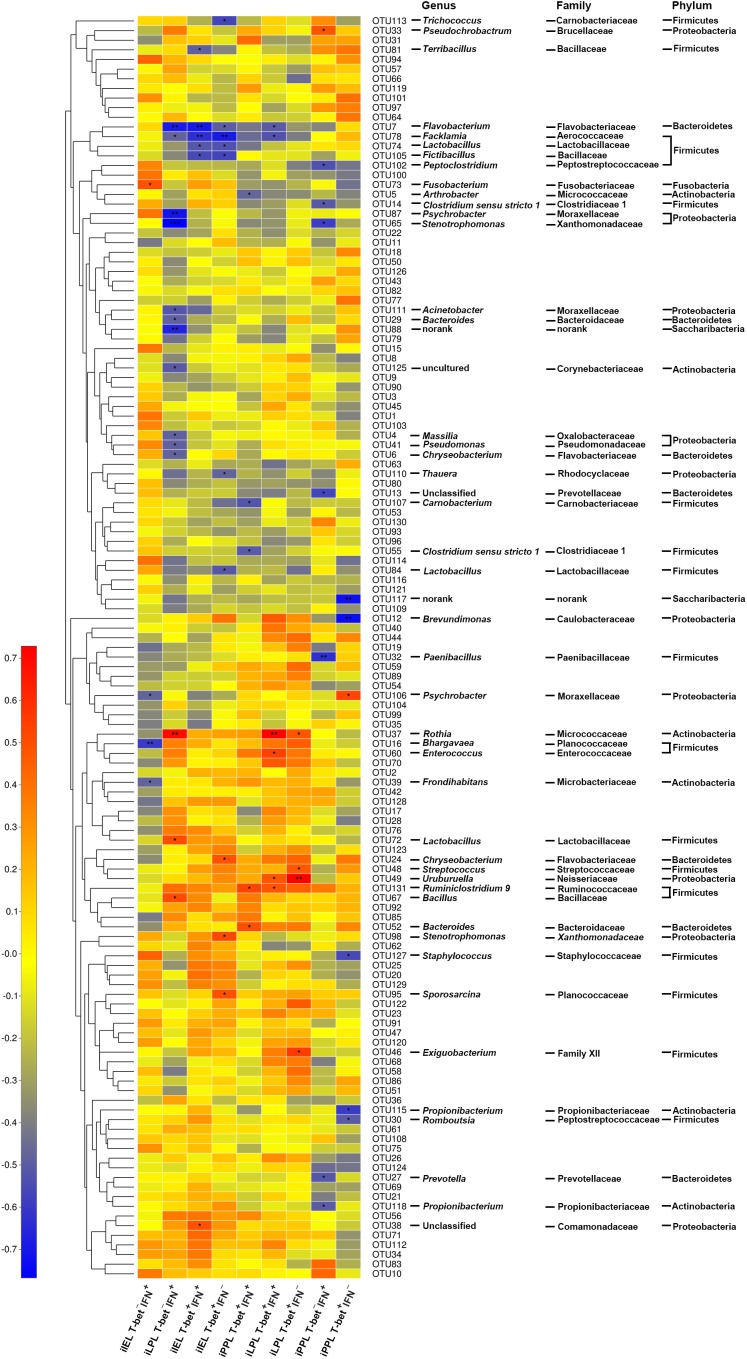
Correlation between OTUs and CD3^-^CD19^-^T-bet^+/-^IFNγ^+/-^ cell subsets in the ileum. The relative abundance of ileal microbiota was assessed for correlations with the levels of T-bet^+/-^IFNγ^+/-^ cells among CD3^-^CD19^-^ cell populations in the small intestine. Rows correspond to OTUs with the IDs and the taxonomy of the OTUs (phylum, family, and genus) is shown on the right. OTUs are clustered to the left based on relative abundance. Columns correspond to CD3^-^CD19^-^T-bet^+/-^IFNγ^+/-^ cell subsets. Colors red and blue denote positive and negative association, respectively. The intensity of the colors represents the degree of association between the relative abundance of OTU and CD3^-^CD19^-^T-bet^+/-^IFNγ^+/-^ cell subsets as assessed by Pearson’s correlations. The black asterisks in the blue/red cells indicate that the associations were significant. ^∗^*P* < 0.05, ^∗∗^*P* < 0.01, ^∗∗∗^*P* < 0.001.

Spearman’s correlation analysis was used to identify specific ileal mucosal bacteria that potentially mediated the beneficial effects of LGG on CD3^-^CD19^-^T-bet^+/-^IFNγ^+/-^ cell subsets in the ileum (Figure 6). In total, 49 OTUs were significantly correlated with at least one cell subset in the ileum. Notably, a total of 12 OTUs were correlated with CD3^-^CD19^-^T-bet^+^IFNγ^+^ (6 OTUs) and CD3^-^CD19^-^T-bet^+^IFNγ^-^ (10 OTUs) cells among iIEL. Five OTUs were negatively correlated and one OTU was positively correlated with CD3^-^CD19^-^T-bet^+^IFNγ^+^ cells among iIEL. Seven OTUs were negatively correlated and three OTU was positively correlated with CD3^-^CD19^-^T-bet^+^IFNγ^-^ cells among iIEL. Among, four OTUs were negatively correlated with both CD3^-^CD19^-^T-bet^+^IFNγ^+^ and CD3^-^CD19^-^T-bet^+^IFNγ^-^ cells among iIEL, including OTU7 (*Flavobacterium frigidarium*) and OTU78 (*Facklamia*) that were enriched by LGG, as well as OTU74 (*Lactobacillus*) and OTU105 (*Fictibacillus*). In addition, OTU81 (*Terribacillus*) and OTU38 (an unclassified genus within the family *Comamonadaceae*) were negatively and positively correlated with CD3^-^CD19^-^T-bet^+^IFNγ^+^ cells among iIEL, respectively. OTU84, OTU110, and OTU113 were negatively and OTU24, OTU95, and OTU98 were positively correlated with CD3^-^CD19^-^T-bet^+^IFNγ^-^ cells among iIEL.

## Discussion

*S.* 4,[5],12:i:- is responsible for increased foodborne salmonellosis outbreaks associated with pig meat. Pigs have been identified as a primary reservoir of serovar 4,[5],12:i:- strains (Hopkins et al., 2010). We previously found that oral inoculation of newly weaned piglets with *S.* 4,[5],12:i:- results in fever and enteritis in the ileum, however, administration of LGG ameliorates the intestinal lesions and prevents the excessive inflammatory response caused by *S.* 4,[5],12:i:- (Yu et al., 2017). Gut microbiota dysbiosis is a leading cause of post-weaning diarrhea in pigs (Gresse et al., 2017). Perturbation of gut microbiota leads to a dysregulated response of innate immune cells and may predispose the host to enteric infection. In the present study, administration of LGG maintains the gut microbiota homeostasis and changes the *Prevotellaceae NK3B31 group*-centric microbiota network caused by *S.* 4,[5],12:i:-. Additionally, administration of LGG affects the proportion of CD3^-^CD19^-^T-bet^+^IFNγ^+^ and CD3^-^CD19^-^T-bet^+^IFNγ^-^ cell subsets to provide defense against *S.* 4,[5],12:i:-.

Gut microbiota provides colonization resistance against invading pathogens involving promoting host tissue development, physiology, and mucosal immunology (Leatham et al., 2009). *Salmonella* has developed some strategies to circumvent gut microbiota-mediated colonization resistance. *S.* Typhimurium exploits inflammation to modify swine intestinal microbiota, which contributes to impaired colonization resistance and in turn increases the host susceptibility to *S.* Typhimurium colonization (Thiennimitr et al., 2011; Drumo et al., 2015). In the present study, clustering analysis showed an aberrant ileal mucosal microbiota pattern in newly weaned piglets challenged with *S.* 4,[5],12:i:-. Some unique taxa, including *Chryseobacterium, Deinococcus, Ruminococcaceae NK4A214 group, Staphylococcus, Propionibacterium*, and uncultured *Pseudomonadaceae* populations, were only found in pigs challenged with *S.* 4,[5],12:i:-, as also demonstrated by Venn and network analysis. Some species belonging to *Chryseobacterium, Staphylococcus*, and *Propionibacterium* have been recognized as important pathogens causing infectionious diseases in infants and children (An et al., 2017; Das et al., 2017; Swarup et al., 2018). Gut microbiota with higher complexity facilitates *S.* Typhimurium clearance (Endt et al., 2010). Heatmap analysis showed that untreated control pigs and pigs pretreated with LGG had a similar microbiota composition. *Fusobacterium* was exclusively present in the pigs pretreated with LGG. Our data indicate LGG restores the gut microbiota balance to defense against *S.* 4,[5],12:i:- infection in pigs.

We observed a clear alteration in the gut microbiota in response to *S.* 4,[5],12:i:-challenge and LGG pretreatment. *S.* 4,[5],12:i:- challenge was associated with increased relative abundances of *Betaproteobacteria* populations, an unclassified *Psychrobacter* genus within *Gammaproteobacteria* and *Chryseobacterium indoltheticum*, as shown by LEfSe analysis. The ANOVA results also showed that *S.* 4,[5],12:i:- challenge led to an elevated level of *Prevotellaceae NK3B31 group, Psychrobacter, Chryseobacterium indoltheticum* and uncultured *Corynebacteriaceae* populations. These specific changes of taxa may contribute to strengthen *S.* 4,[5],12:i:- infection. Administration of LGG attenuated the increased populations of *Prevotellaceae NK3B31 group* and uncultured *Corynebacteriaceae* caused by *S.* 4,[5],12:i:-. *Flavobacterium frigidarium* was also enriched in the pigs pretreated with LGG. In line with these observations, *Corynebacteriaceae* was positively and *Flavobacterium* was negatively correlated with skin inflammation in ovine footrot (Maboni et al., 2017).

Correlation network analysis revealed that there were more correlations and more clustering of OTUs in pigs challenged with *S.* 4,[5],12:i:-, compared with untreated control pigs and pigs pretreated with LGG. The betweenness centrality was lower in the SM network, indicating there were more OTUs that were highly connected to other OTUs in pigs challenged with *S.* 4,[5],12:i:-. It is speculated that the altered network structure in pigs challenged with *S.* 4,[5],12:i:- may be involved in the increased susceptibility to *S.* 4,[5],12:i:- infection. LGG administration enables reprogramming of microbe–microbe interactions to provide defense against *S.* 4,[5],12:i:- infection.

To further explored the biological significance of microbe–microbe interactions, we identified the co-occurrence patterns of all observed OTUs across all pigs. A total of 114 positive correlations and 18 negative correlations were found. The relationship between OTUs was largely drove by *S.* 4,[5],12:i:- challenge or LGG pretreatment. A *Prevotellaceae NK3B31 group*-centric co-occurrence sub-network was shaped by *S.* 4,[5],12:i:-. *Prevotellaceae NK3B31 group* enriched in pigs challenged with *S.* 4,[5],12:i:- was positively correlated with *Chryseobacterium* and an uncultured genus within the family *Corynebacteriaceae*, which was unique and significantly increased in pigs challenged with *S.* 4,[5],12:i:-, respectively. *Prevotellaceae NK3B31 group* was also positively correlated with *Acinetobacter*. Some multidrug-resistant *Acinetobacter* species rank among the most important nosocomial pathogens (Dijkshoorn et al., 2007). *Fusobacterium* which was specific to the pigs pretreated with LGG was positively correlated with *Lactobacillus animalis* and *Propionibacterium*. *Lactobacillus animalis* can regulate immune response and influence composition and metabolism of the intestinal microflora to control infections (Biagi et al., 2007; Karunasena et al., 2013). Our data suggested that *S.* 4,[5],12:i:- challenge forms a *Prevotellaceae NK3B31 group*-centric species correlation network and this ecological relationship provides *S.* 4,[5],12:i:- with a competitive advantage to cause intestinal inflammation. LGG deliberately restores gut microbiota through attenuation of the increase in the population of *Prevotellaceae NK3B31 group* caused by *S.* 4,[5],12:i:- and promotion of symbiotic synergism of *Fusobacterium, Lactobacillus animalis*, and *Propionibacterium*. We postulate that this remolded gut microbiota are associated with LGG prevention of *S.* 4,[5],12:i:- infection.

Targeting of the non-B-non-T CD3^-^CD19^-^ cell subsets, the innate counterparts of adaptive T cells, facilitate an early immune response to pathogens via modulation of cytokine function along with preventive strategies. LGG administration induced the expansion of CD3^-^CD19^-^T-bet^+^IFNγ^+^ cells and CD3^-^CD19^-^T-bet^+^IFNγ^-^ in the peripheral blood at 24 h after *S.* 4,[5],12:i:- infection. Concurrent with our results, T-bet upregulation is observed in immunosuppressed mice with *L. plantarum* NCU116 (Xie et al., 2015). T-bet-dependent IFNγ function in some CD3^-^CD19^-^ cell subsets plays a critical role early in the immune response to pathogens. For example, genomic T-bet deficiency renders mice highly susceptible to colitis, and adoptive transfer of ILC1s to lymphocyte-deficient mice is sufficient to boost immunity (Klose et al., 2014). Graded expression of T-bet in CCR6^-^RORγt^+^ ILCs facilitates the differentiation of IFNγ-producing CCR6^-^RORγt^+^ ILCs required to protect the epithelial barrier against *Salmonella* infections (Klose et al., 2013), implying that T-bet is indispensable for the functional plasticity of some groups in CD3^-^CD19^-^ cell subsets against pathogens. Early IFNγ production in human blood controls intracellular *S.* Typhimurium infections and reduces the threat of the extracellular spread of the disease along with bacteremia (Nyirenda et al., 2010). IFNγ is vital for controlling various pathogenic infections early after infection and may restrict *S.* Typhimurium infection by limiting the availability of iron for intracellular *S.* Typhimurium or triggering the death of infected cells (Ingram et al., 2018). The present data suggest that LGG administration may promote early immunity to *S.* 4,[5],12:i:- infection by inducing the expansion of CD3^-^CD19^-^T-bet^+^IFNγ^+^ and CD3^-^CD19^-^T-bet^+^IFNγ^-^ cell subsets in the peripheral blood.

Whereas acute inflammation is a necessary process to protect against enteric infection, chronic inflammation directly contributes to the pathogenesis and progression of multiple infectious and inflammatory disorders. In the present study, an *S.* 4,[5],12:i:- challenge increased the proportion of intraepithelial CD3^-^CD19^-^T-bet^+^IFNγ^+^ and CD3^-^CD19^-^T-bet^+^IFNγ^-^ cell subsets in the ileum; however, LGG administration attenuated this increase. Mice deficient in IFNγ exhibit markedly attenuated intestinal inflammation during *S.* Typhimurium infection (Rhee et al., 2005; Awoniyi et al., 2012). In fate-mapping mouse models, infection with *S.* Typhimurium results in the downregulation of RORγt in the ILCs in the CD3^-^CD19^-^ cell subset; these ILCs resemble ILC1s expressing T-bet and IFNγ, thus promoting intestinal inflammation (Powell et al., 2012). Intraepithelial ILC1s and IFNγ-producing ILC3s accumulated in mice, and blocking IFNγ ameliorated intestinal inflammation in a mouse model of colitis (Buonocore et al., 2010). These results indicate that LGG administration attenuates the increase in the proportion of intraepithelial CD3^-^CD19^-^T-bet^+^IFNγ^+^ cells and CD3^-^CD19^-^T-bet^+^IFNγ^-^ cells owing to *S.* 4,[5],12:i:- in the ileum, potentially aiding the amelioration of chronic intestinal inflammation.

Aberrations in the communication between the gut microbiota and the innate immune system may contribute to inflammatory disorders. Correlation analysis revealed that six and ten phylotypes were correlated with intraepithelial CD3^-^CD19^-^T-bet^+^IFNγ^+^ and CD3^-^CD19^-^T-bet^+^IFNγ^-^ cells in the ileum, respectively. Both *Flavobacterium frigidarium* and *Facklamia* that were increased by LGG were negatively correlated with intraepithelial CD3^-^CD19^-^T-bet^+^IFNγ^+^ and CD3^-^CD19^-^T-bet^+^IFNγ^-^ cells. Down-regulation of the ileum inflammatory response by sodium butyrate intervention is following the increased relative abundance of *Facklamia* in neonatal piglets (Xu et al., 2016). Other phylotypes that were negatively correlated with intraepithelial CD3^-^CD19^-^T-bet^+^IFNγ^+^ and CD3^-^CD19^-^T-bet^+^IFNγ^-^ cells may be potentially beneficial bacteria, including *Fictibacillus, Terribacillus, Trichococcus*, and *Thauera*. Meanwhile, those phylotypes that were positively correlated with intraepithelial CD3^-^CD19^-^T-bet^+^IFNγ^+^ and CD3^-^CD19^-^T-bet^+^IFNγ^-^ cells may consist of potentially harmful bacteria. For example, *Chryseobacterium* were enriched in individuals with IBD or neonatal respiratory tract infection (Suchodolski et al., 2010; Virok et al., 2014). The present data suggest a functional and ecologically plausible negative impact of the specific members of the microbiota in CD3^-^CD19^-^ cell subsets. LGG probably enriches *Flavobacterium frigidarium* and *Facklamia*, which in turn attenuates the expansion of intraepithelial CD3^-^CD19^-^T-bet^+^IFNγ^+^ and CD3^-^CD19^-^T-bet^+^IFNγ^-^ cell subsets in the ileum, thereby restricting the chronic intestinal inflammation caused by *S.* 4,[5],12:i:-.

Several concerns need to be addressed in further studies. Oral administration of probiotic LGG does not significantly improve *Salmonella* infection-induced fever and enteritis in piglets, possibly due to the short lasting time. Further study needs more experimental piglets and longer lasting time to determine if potential recovery is possible. The limitations of the present study include the use of some anti-human antibodies to target the markers in pigs. It is necessary to correctly estimate the true frequency of marker proteins by preparing and purifying the anti-pig antibodies. It is also essential to discriminate different CD3^-^CD19^-^ cell subsets through precise targeting of the lineage marker mix and to identify which cell subset plays a central role in LGG-mediated control of *S.* 4,[5],12:i:- infection. In studies investigating the potential role of gut microbiota in health or disease, when a correlation is established between certain microbes and a specific disease condition, further studies along with those investigating simple associations are required to establish the mechanistic link between causative strains to phenotypes and show the causality as a proof of concept.

## Conclusion

**FIGURE 7:**
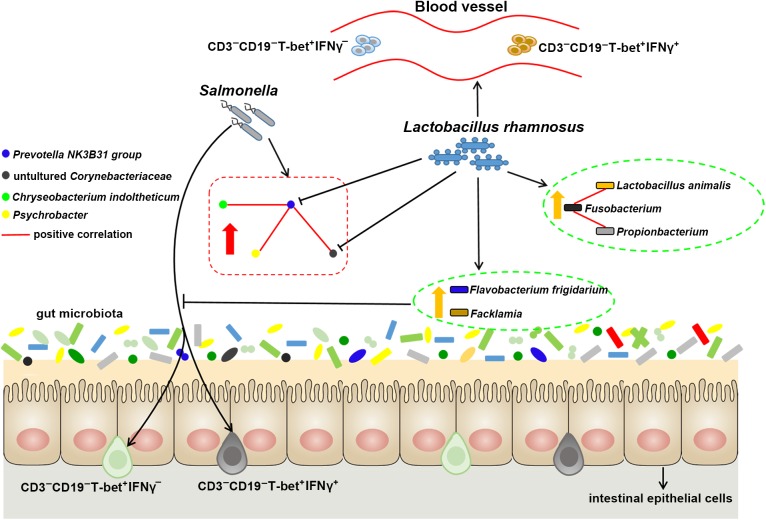
Probiotic LGG alters ileal microbiota with associated CD3^-^CD19^-^T-bet^+^IFNγ^+/-^ cell subset homeostasis in pigs. *S.* 4,[5],12:i:- challenge led to disturbed gut microbiota, characterized by increased levels of *Psychrobacter, Chryseobacterium indoltheticum*, and uncultured *Corynebacteriaceae* populations, as well as an aberrant correlation network in *Prevotellaceae NK3B31 group*-centric species. LGG reduces the population of *Prevotellaceae NK3B31 group*, changes the correlation network in *Prevotellaceae NK3B31 group*-centric species, and promotes symbiotic synergism of *Fusobacterium, Lactobacillus animalis*, and *Propionibacterium*. LGG attenuates the increase in the population of intraepithelial CD3^-^CD19^-^T-bet^+^IFNγ^+^ and CD3^-^CD19^-^T-bet^+^IFNγ^-^ cells resulting from an *S.* 4,[5],12:i:- infection in the ileum. LGG enriched *Flavobacterium frigidarium* and *Facklamia* populations, which are negatively correlated with intraepithelial CD3^-^CD19^-^T-bet^+^IFNγ^+^ and CD3^-^CD19^-^T-bet^+^IFNγ^-^ cells in the ileum.

In conclusion, the present suggest that after *S.* 4,[5],12:i:- infection, LGG administration enables reprogramming of microbe–microbe interactions and alters ileal microbiota with associated specific CD3^-^CD19^-^ cell subset homeostasis (Figure 7). LGG reduces the cell population in the *Prevotellaceae NK3B31 group*, changes the correlation network in *Prevotellaceae NK3B31 group*-centric species, and promotes symbiotic synergism of *Fusobacterium, Lactobacillus animalis*, and *Propionibacterium*. LGG attenuates the increase in the population of intraepithelial CD3^-^CD19^-^T-bet^+^IFNγ^+^ and CD3^-^CD19^-^T-bet^+^IFNγ^-^ cell subsets resulting from an *S.* 4,[5],12:i:- infection in the ileum. LGG enriched *Flavobacterium frigidarium* and *Facklamia* populations, which are negatively correlated with intraepithelial CD3^-^CD19^-^T-bet^+^IFNγ^+^ and CD3^-^CD19^-^T-bet^+^IFNγ^-^ cells in the ileum.

## Author Contributions

WZ, QW, HJ, and JW conceived and designed the experiments and wrote the manuscript. WZ, QW, YZ, GY, and JY performed the experiments. WZ and QW performed high-through sequencing analysis. YZ, GY, and WZ performed the flow cytometry analysis.

## Conflict of Interest Statement

The authors declare that the research was conducted in the absence of any commercial or financial relationships that could be construed as a potential conflict of interest.

## Footnotes

1 http://qiime.org

2 www.arb-silva.de/

3 http://sourceforge.net/projects/rdp-classifier

4 http://www.mothur.org

5 www.i-sanger.com

6 http://www.cytoscape.org/

7 https://www.r-project.org/

## Supplementary Material

FIGURE S1 Alpha diversity comparison. Ileal mucosal microbiota diversity was estimated using the rarefaction abundance curves **(A)**, Shannon diversity curves **(B)**, and rank abundance curves **(C)** at 97% similarity (*n* = 6 per group) that received oral sterile physiological saline (CN), oral sterile physiological saline followed by *S.* 4,[5],12:i:- (1 × 10^10^ CFU/ml, 10 ml) challenge (SM), or LGG (1 × 10^9^ CFU/ml, 10 ml/day) for 1 week followed by *S.* 4,[5],12:i:- (LS). CN (C), SM (S) or LS (L) plus a digit (1–6) represents an individual pig in the corresponding groups.

FIGURE S2 Gating strategy for cell analysis by flow cytometry in the peripheral blood. **(A)** FSC/SSC dot plot of peripheral blood lymphocytes, cells with no gating. CD3/CD19 dot plot, cells were gated on lymphocytes. T-bet/IFNγ dot plot, cells were gated on CD3^-^CD19^-^. **(B)** Each marker was gated through negative and isotype control staining.

FIGURE S3 Gating strategy for cell analysis by flow cytometry in the intestines. **(A)** FSC/SSC dot plot of intestinal lymphocytes, cells with no gating. CD3/CD19 dot plot, cells were gated on lymphocytes. T-bet/IFNγ dot plot, cells were gated on CD3^-^CD19^-^. **(B)** Each marker was gated through negative and isotype control staining.

TABLE S1 Relative abundance (%) of the predominant taxa in the ileal mucosal microbiota of pigs.

TABLE S2 ADONIS analysis based on Bray–Curtis distance at different taxonomic levels.

TABLE S3 Properties of correlation networks generated from samples from CN, SM, and LS.

TABLE S4 Pearson’s correlations or associations between the all OTUs with an absolute Pearson’s correlation above 0.7.
