# Supplementary figures and images for: Probiotic Lactobacillus rhamnosus GG Induces Alterations in Ileal Microbiota With Associated CD3-CD19-T-bet+IFNγ+/- Cell Subset Homeostasis in Pigs Challenged With Salmonella enterica Serovar 4,[5],12:i:-
Source: Front Microbiol. 2019 May 7;10:977. doi: 10.3389/fmicb.2019.00977 (PMC6516042; doi:10.3389/fmicb.2019.00977)

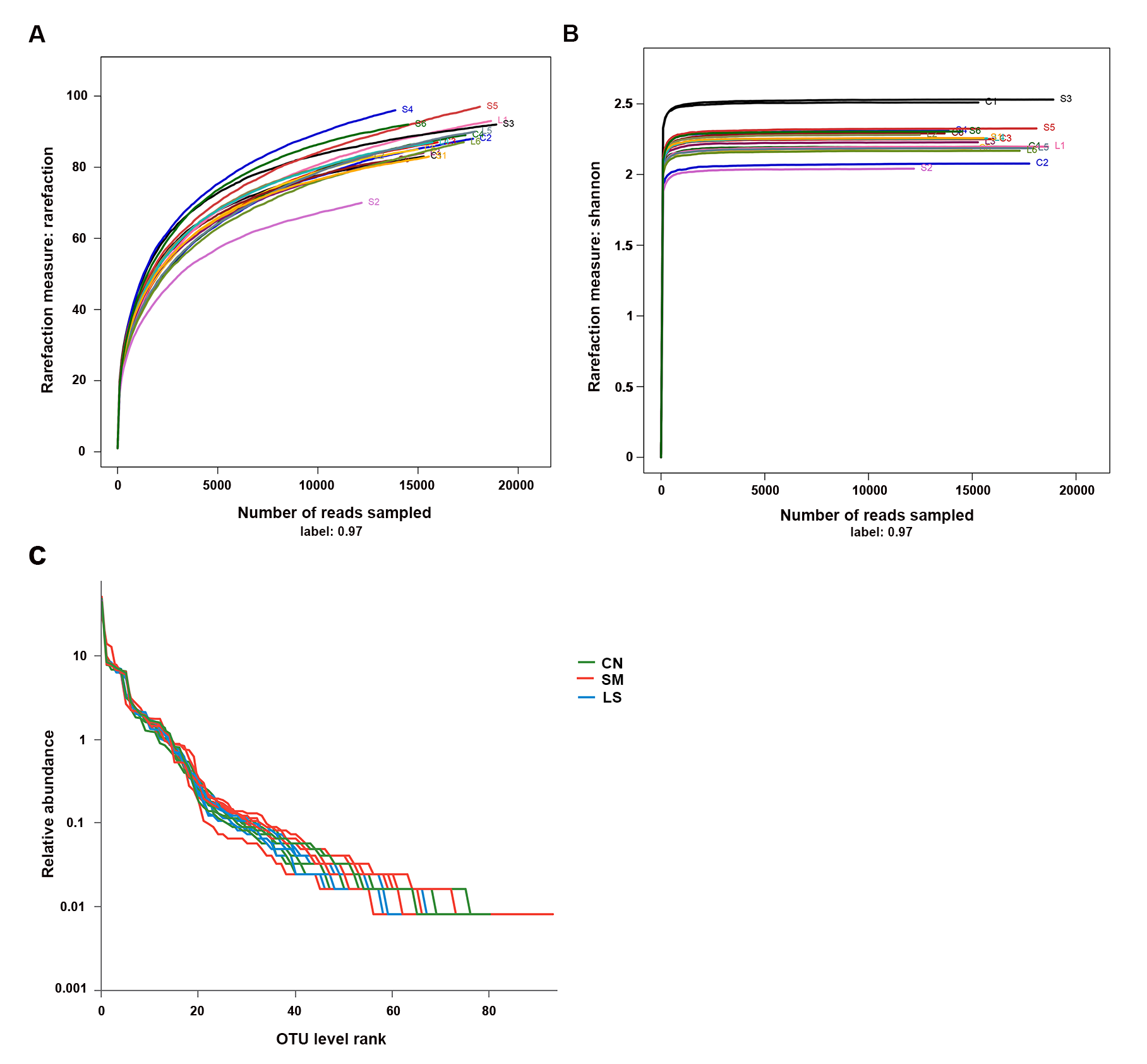

Supplement: FIGURE S1 — Alpha diversity comparison. Ileal mucosal microbiota diversity was estimated using the rarefaction abundance curves (A), Shannon diversity curves (B), and rank abundance curves (C) at 97% similarity (n = 6 per group) that received oral sterile physiological saline (CN), oral sterile physiological saline followed by S. 4,[5],12:i:- (1 × 10^10^ CFU/ml, 10 ml) challenge (SM), or LGG (1 × 10^9^ CFU/ml, 10 ml/day) for 1 week followed by S. 4,[5],12:i:- (LS). CN (C), SM (S) or LS (L) plus a digit (1–6) represents an individual pig in the corresponding groups. [file Image_1.TIF]

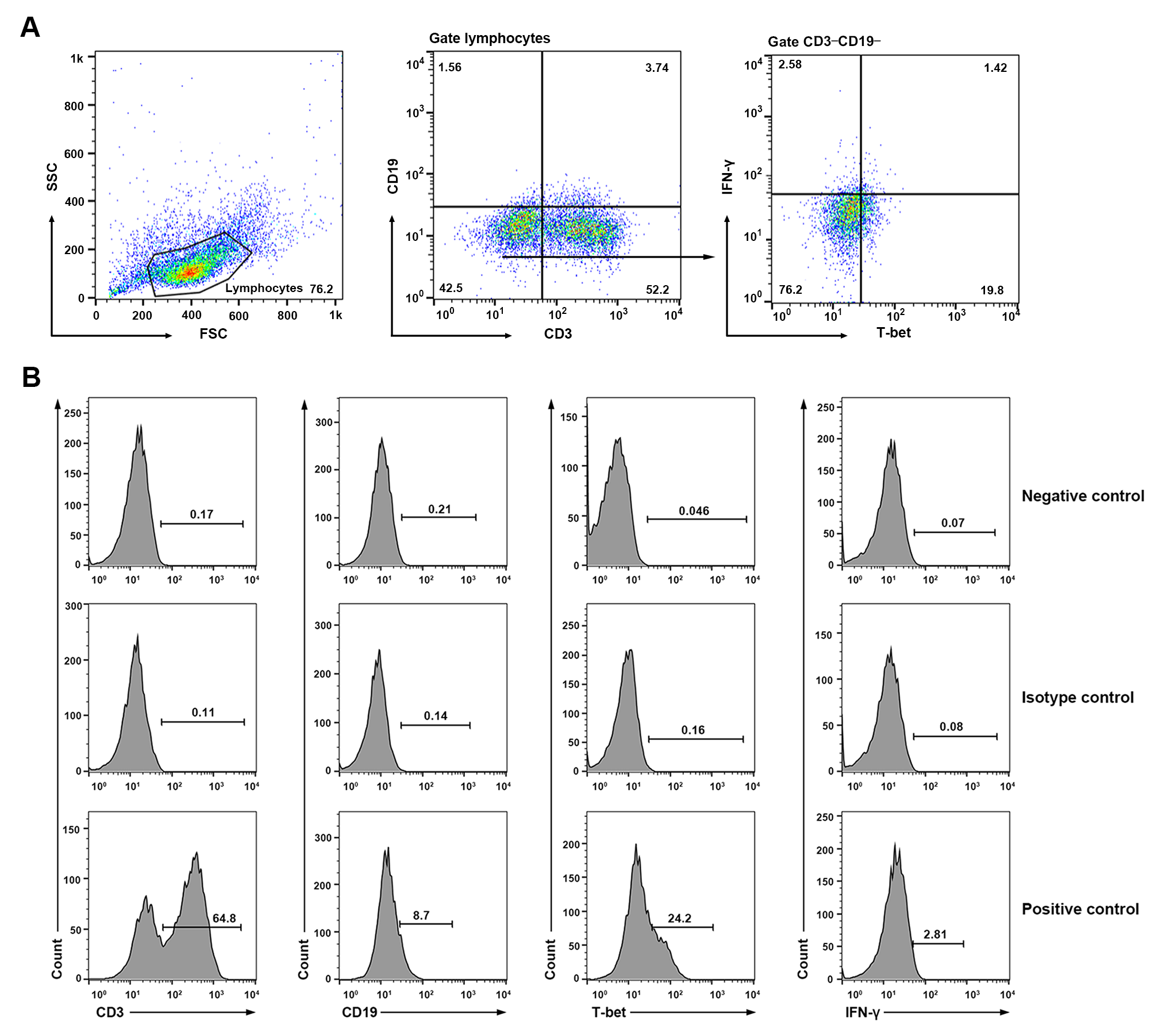

Supplement: FIGURE S2 — Gating strategy for cell analysis by flow cytometry in the peripheral blood. (A) FSC/SSC dot plot of peripheral blood lymphocytes, cells with no gating. CD3/CD19 dot plot, cells were gated on lymphocytes. T-bet/IFNγ dot plot, cells were gated on CD3-CD19-. (B) Each marker was gated through negative and isotype control staining. [file Image_2.TIF]

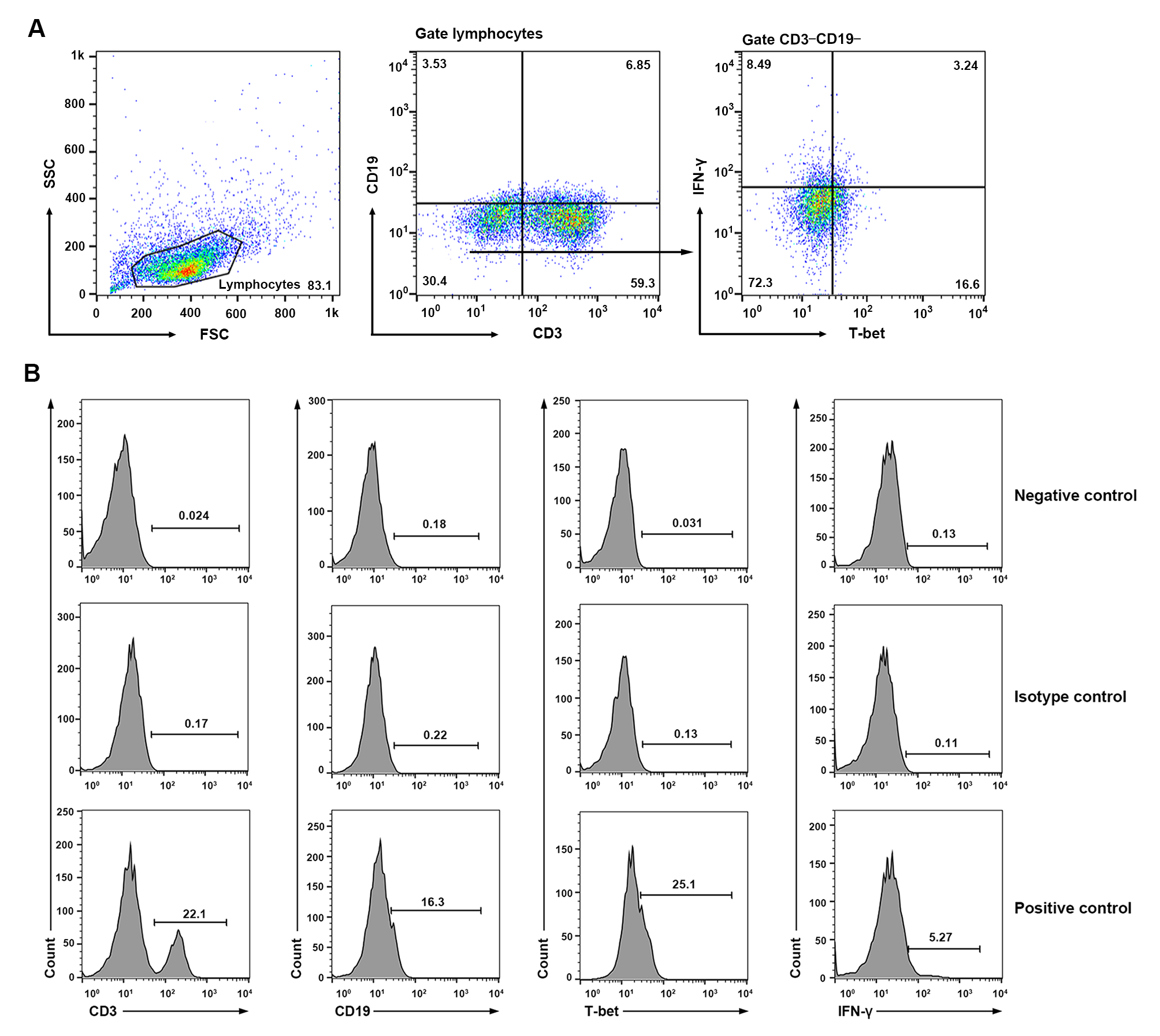

Supplement: FIGURE S3 — Gating strategy for cell analysis by flow cytometry in the intestines. (A) FSC/SSC dot plot of intestinal lymphocytes, cells with no gating. CD3/CD19 dot plot, cells were gated on lymphocytes. T-bet/IFNγ dot plot, cells were gated on CD3-CD19-. (B) Each marker was gated through negative and isotype control staining. [file Image_3.TIF]
