# Supplementary material for: Probiotic Lactobacillus rhamnosus GG Induces Alterations in Ileal Microbiota With Associated CD3-CD19-T-bet+IFNγ+/- Cell Subset Homeostasis in Pigs Challenged With Salmonella enterica Serovar 4,[5],12:i:-
Source: Front Microbiol. 2019 May 7;10:977. doi: 10.3389/fmicb.2019.00977 (PMC6516042; doi:10.3389/fmicb.2019.00977)
Supplement: TABLE S1 — Relative abundance (%) of the predominant taxa in the ileal mucosal microbiota of pigs. [file Table_1.DOC]

**TABLE S1. Relative abundance (%) of the predominant taxa in the ileal mucosal microbiota of pigs.**

|  |  |  | **Treatment^a^** | | |
| --- | --- | --- | --- | --- | --- |
| **OTU** | **Genus** | **Species** | **CN** | **SM** | **LS** |
| OTU57 | *Lactococcus* | *Lactococcus piscium* | 45.08 | 42.33 | 45.23 |
| OTU66 | *Lactococcus* | Unclassified | 8.22 | 7.72 | 8.31 |
| OTU18 | *Solibacillus* | *Solibacillus silvestris* | 7.73 | 8.77 | 8.02 |
| OTU85 | *Bacillus* | Unclassified | 6.49 | 6.35 | 6.25 |
| OTU19 | *Bacillus* | Unclassified | 6.36 | 7.33 | 6.53 |
| OTU108 | *Arthrobacter* | Unclassified | 6.08 | 5.89 | 5.17 |
| OTU86 | *Pseudomonas* | Unclassified | 2.60 | 2.68 | 2.75 |
| OTU67 | *Bacillus* | Unclassified | 2.15 | 2.03 | 2.05 |
| OTU68 | *Pseudomonas* | *Pseudomonas gessardii* | 1.83 | 2.17 | 2.03 |
| OTU41 | *Pseudomonas* | Unclassified | 1.75 | 1.98 | 1.98 |
| OTU89 | *Lysinibacillus* | *Lysinibacillus xylanilyticus* | 1.49 | 1.55 | 1.47 |
| OTU50 | *Bacillus* | *Bacillus oceanisediminis* | 1.45 | 1.53 | 1.47 |
| OTU46 | *Exiguobacterium* | *Exiguobacterium sp. AT1b* | 1.17 | 1.39 | 1.16 |
| OTU107 | *Carnobacterium* | *Carnobacterium maltaromaticum* | 1.13 | 1.04 | 1.16 |

^a^Pigs received sterile physiological saline orally (CN), received sterile physiological saline orally followed by *S.* 4,[5],12:i:- challenge (1 × 10^10^ CFU/ml, 10 ml, per os [p.o.]) (SM), were intragastrically administrated of LGG (1 × 10^9^ CFU/ml, 10 ml once daily) for 1 week followed by *S.* 4,[5],12:i:- challenge (LS).
