# Supplementary material for: Probiotic Lactobacillus rhamnosus GG Induces Alterations in Ileal Microbiota With Associated CD3-CD19-T-bet+IFNγ+/- Cell Subset Homeostasis in Pigs Challenged With Salmonella enterica Serovar 4,[5],12:i:-
Source: Front Microbiol. 2019 May 7;10:977. doi: 10.3389/fmicb.2019.00977 (PMC6516042; doi:10.3389/fmicb.2019.00977)
Supplement: TABLE S2 — ADONIS analysis based on Bray–Curtis distance at different taxonomic levels. [file Table_2.docx]

**TABLE S2. ADONIS analysis based on Bray-Curtis distance at different taxonomic levels.**

| Taxonomic level | *R*^2^ value | *P* value |
| --- | --- | --- |
| phylum | 0.37 | 0.018 |
| class | 0.35 | 0.018 |
| order | 0.31 | 0.013 |
| family | 0.24 | 0.011 |
| genus | 0.24 | 0.018 |
| OTU | 0.23 | 0.014 |
