# Supplementary material for: Probiotic Lactobacillus rhamnosus GG Induces Alterations in Ileal Microbiota With Associated CD3-CD19-T-bet+IFNγ+/- Cell Subset Homeostasis in Pigs Challenged With Salmonella enterica Serovar 4,[5],12:i:-
Source: Front Microbiol. 2019 May 7;10:977. doi: 10.3389/fmicb.2019.00977 (PMC6516042; doi:10.3389/fmicb.2019.00977)
Supplement: TABLE S3 — Properties of correlation networks generated from samples from CN, SM, and LS. [file Table_3.DOCX]

**TABLE S3. Properties of correlation networks generated from samples from CN, SM and LS.**

| **parameter** | **treatment** | | |
| --- | --- | --- | --- |
|  | **CN** | **SM** | **LS** |
| Nodes | 118 | 120 | 117 |
| Edges | 416 | 679 | 353 |
| Mean degree | 7.05 | 11.32 | 6.03 |
| Transitivity | 0.604 | 0.769 | 0.577 |
| Average path length | 4.52 | 6.04 | 4.52 |
| Betweeness centrality | 0.023 | 0.043 | 0.031 |
| Closeness centrality | 0.253 | 0.182 | 0.227 |
| Positive correlation number | 555 | 881 | 483 |
| Negative correlation number | 337 | 320 | 316 |
