# Supplementary material for: Probiotic Lactobacillus rhamnosus GG Induces Alterations in Ileal Microbiota With Associated CD3-CD19-T-bet+IFNγ+/- Cell Subset Homeostasis in Pigs Challenged With Salmonella enterica Serovar 4,[5],12:i:-
Source: Front Microbiol. 2019 May 7;10:977. doi: 10.3389/fmicb.2019.00977 (PMC6516042; doi:10.3389/fmicb.2019.00977)
Supplement: TABLE S4 — Pearson’s correlations or associations between the all OTUs with an absolute Pearson’s correlation above 0.7. [file Table_4.DOCX]

**TABLE S4. Pearson’s correlations or associations between the all OTUs with an absolute Pearson’s correlation above 0.7.**

| **OTU A** | **OTU B** | **Pearson correlation** | ***P* value** |
| --- | --- | --- | --- |
| OTU111 | OTU121 | 0.999 | 0 |
| OTU21 | OTU20 | 0.997 | 0 |
| OTU19 | OTU18 | 0.979 | 0 |
| OTU28 | OTU21 | 0.969 | 0 |
| OTU21 | OTU27 | 0.967 | 0 |
| OTU128 | OTU123 | 0.966 | 0 |
| OTU19 | OTU4 | 0.966 | 0 |
| OTU28 | OTU20 | 0.965 | 0 |
| OTU18 | OTU4 | 0.964 | 0 |
| OTU27 | OTU20 | 0.962 | 0 |
| OTU28 | OTU27 | 0.931 | 0 |
| OTU121 | OTU115 | 0.929 | 0 |
| OTU111 | OTU115 | 0.923 | 0 |
| OTU30 | OTU38 | 0.907 | 0 |
| OTU79 | OTU77 | 0.904 | 0 |
| OTU25 | OTU125 | 0.891 | 0 |
| OTU30 | OTU55 | 0.890 | 0 |
| OTU26 | OTU28 | 0.877 | 0 |
| OTU18 | OTU51 | 0.875 | 0 |
| OTU18 | OTU44 | 0.875 | 0 |
| OTU77 | OTU118 | 0.874 | 0 |
| OTU108 | OTU96 | 0.872 | 0 |
| OTU38 | OTU55 | 0.871 | 0 |
| OTU19 | OTU44 | 0.870 | 0 |
| OTU30 | OTU129 | 0.866 | 0 |
| OTU44 | OTU51 | 0.861 | 0 |
| OTU57 | OTU66 | 0.858 | 0 |
| OTU26 | OTU21 | 0.856 | 0 |
| OTU4 | OTU109 | 0.850 | 0 |
| OTU9 | OTU19 | 0.850 | 0 |
| OTU26 | OTU20 | 0.846 | 0 |
| OTU50 | OTU89 | 0.846 | 0 |
| OTU9 | OTU4 | 0.838 | 1.00E-06 |
| OTU4 | OTU51 | 0.835 | 1.00E-06 |
| OTU126 | OTU43 | 0.832 | 1.00E-06 |
| OTU44 | OTU4 | 0.827 | 1.00E-06 |
| OTU25 | OTU59 | 0.825 | 1.00E-06 |
| OTU25 | OTU115 | 0.822 | 2.00E-06 |
| OTU19 | OTU51 | 0.819 | 2.00E-06 |
| OTU38 | OTU129 | 0.816 | 2.00E-06 |
| OTU9 | OTU18 | 0.814 | 3.00E-06 |
| OTU25 | OTU111 | 0.814 | 3.00E-06 |
| OTU62 | OTU129 | 0.809 | 4.00E-06 |
| OTU25 | OTU121 | 0.806 | 4.00E-06 |
| OTU88 | OTU19 | 0.804 | 5.00E-06 |
| OTU88 | OTU18 | 0.803 | 5.00E-06 |
| OTU39 | OTU4 | 0.802 | 5.00E-06 |
| OTU4 | OTU42 | 0.802 | 5.00E-06 |
| OTU39 | OTU42 | 0.801 | 5.00E-06 |
| OTU88 | OTU4 | 0.800 | 6.00E-06 |
| OTU26 | OTU27 | 0.798 | 6.00E-06 |
| OTU124 | OTU54 | 0.798 | 7.00E-06 |
| OTU8 | OTU19 | 0.798 | 7.00E-06 |
| OTU122 | OTU42 | 0.797 | 7.00E-06 |
| OTU68 | OTU51 | 0.795 | 8.00E-06 |
| OTU55 | OTU129 | 0.792 | 9.00E-06 |
| OTU59 | OTU125 | 0.791 | 9.00E-06 |
| OTU8 | OTU4 | 0.791 | 9.00E-06 |
| OTU19 | OTU42 | 0.788 | 1.10E-05 |
| OTU39 | OTU19 | 0.786 | 1.20E-05 |
| OTU38 | OTU62 | 0.782 | 1.50E-05 |
| OTU42 | OTU109 | 0.778 | 1.80E-05 |
| OTU112 | OTU121 | 0.778 | 1.80E-05 |
| OTU46 | OTU58 | 0.777 | 1.90E-05 |
| OTU25 | OTU127 | 0.771 | 2.40E-05 |
| OTU44 | OTU42 | 0.771 | 2.50E-05 |
| OTU88 | OTU51 | 0.768 | 2.80E-05 |
| OTU51 | OTU41 | 0.765 | 3.20E-05 |
| OTU30 | OTU62 | 0.765 | 3.30E-05 |
| OTU112 | OTU111 | 0.764 | 3.40E-05 |
| OTU123 | OTU47 | 0.763 | 3.50E-05 |
| OTU111 | OTU103 | 0.763 | 3.60E-05 |
| OTU8 | OTU18 | 0.760 | 4.00E-05 |
| OTU88 | OTU44 | 0.760 | 4.10E-05 |
| OTU112 | OTU115 | 0.758 | 4.40E-05 |
| OTU103 | OTU121 | 0.757 | 4.50E-05 |
| OTU19 | OTU109 | 0.757 | 4.50E-05 |
| OTU8 | OTU122 | 0.756 | 4.70E-05 |
| OTU8 | OTU39 | 0.756 | 4.70E-05 |
| OTU126 | OTU18 | 0.756 | 4.80E-05 |
| OTU88 | OTU9 | 0.755 | 5.00E-05 |
| OTU26 | OTU12 | 0.752 | 5.60E-05 |
| OTU39 | OTU18 | 0.750 | 6.00E-05 |
| OTU18 | OTU42 | 0.749 | 6.20E-05 |
| OTU51 | OTU109 | 0.746 | 7.10E-05 |
| OTU39 | OTU109 | 0.742 | 8.10E-05 |
| OTU18 | OTU109 | 0.739 | 9.40E-05 |
| OTU79 | OTU118 | 0.738 | 9.60E-05 |
| OTU125 | OTU115 | 0.736 | 1.04E-04 |
| OTU126 | OTU51 | 0.734 | 1.12E-04 |
| OTU54 | OTU59 | 0.728 | 1.38E-04 |
| OTU126 | OTU19 | 0.725 | 1.53E-04 |
| OTU68 | OTU44 | 0.725 | 1.54E-04 |
| OTU101 | OTU61 | 0.724 | 1.56E-04 |
| OTU9 | OTU39 | 0.723 | 1.62E-04 |
| OTU9 | OTU109 | 0.721 | 1.75E-04 |
| OTU126 | OTU88 | 0.719 | 1.87E-04 |
| OTU13 | OTU14 | 0.718 | 1.94E-04 |
| OTU126 | OTU4 | 0.717 | 2.04E-04 |
| OTU8 | OTU9 | 0.716 | 2.09E-04 |
| OTU128 | OTU47 | 0.713 | 2.30E-04 |
| OTU9 | OTU44 | 0.712 | 2.37E-04 |
| OTU122 | OTU87 | 0.711 | 2.45E-04 |
| OTU68 | OTU9 | 0.710 | 2.54E-04 |
| OTU127 | OTU115 | 0.710 | 2.57E-04 |
| OTU19 | OTU89 | 0.709 | 2.60E-04 |
| OTU112 | OTU84 | 0.709 | 2.65E-04 |
| OTU122 | OTU19 | 0.708 | 2.68E-04 |
| OTU32 | OTU110 | 0.707 | 2.79E-04 |
| OTU68 | OTU89 | 0.707 | 2.82E-04 |
| OTU9 | OTU89 | 0.706 | 2.91E-04 |
| OTU68 | OTU58 | 0.705 | 3.01E-04 |
| OTU124 | OTU127 | 0.704 | 3.06E-04 |
| OTU9 | OTU42 | 0.703 | 3.14E-04 |
| OTU88 | OTU57 | -0.713 | 2.30E-04 |
| OTU44 | OTU66 | -0.716 | 2.07E-04 |
| OTU39 | OTU57 | -0.718 | 1.94E-04 |
| OTU19 | OTU66 | -0.720 | 1.81E-04 |
| OTU122 | OTU66 | -0.730 | 1.30E-04 |
| OTU126 | OTU57 | -0.730 | 1.29E-04 |
| OTU89 | OTU57 | -0.735 | 1.07E-04 |
| OTU8 | OTU57 | -0.746 | 7.00E-05 |
| OTU9 | OTU57 | -0.754 | 5.10E-05 |
| OTU42 | OTU66 | -0.756 | 4.80E-05 |
| OTU46 | OTU66 | -0.776 | 2.00E-05 |
| OTU42 | OTU57 | -0.777 | 1.90E-05 |
| OTU51 | OTU57 | -0.802 | 5.00E-06 |
| OTU57 | OTU46 | -0.827 | 1.00E-06 |
| OTU44 | OTU57 | -0.829 | 1.00E-06 |
| OTU4 | OTU57 | -0.844 | 0 |
| OTU19 | OTU57 | -0.881 | 0 |
| OTU18 | OTU57 | -0.884 | 0 |
